# [μ-Bis(di-*o*-tolyl­phosphan­yl)methane-1:2κ^2^ *P*:*P*′]nona­carbonyl-1κ^3^ *C*,2κ^3^ *C*,3κ^3^ *C*-[diphen­yl(phenyl­sulfanylmeth­yl)phosphane-3κ*P*]-*triangulo*-triruthenium(0) dichloro­methane 0.25-solvate

**DOI:** 10.1107/S160053681100081X

**Published:** 2011-01-15

**Authors:** Omar bin Shawkataly, Imthyaz Ahmed Khan, H. A. Hafiz Malik, Chin Sing Yeap, Hoong-Kun Fun

**Affiliations:** a Chemical Sciences Programme, School of Distance Education, Universiti Sains Malaysia, 11800 USM, Penang, Malaysia; b X-ray Crystallography Unit, School of Physics, Universiti Sains Malaysia, 11800 USM, Penang, Malaysia

## Abstract

In the title compound, [Ru_3_(C_29_H_30_P_2_)(C_19_H_17_PS)(CO)_9_]·0.25CH_2_Cl_2_, the atoms of the dichloro­methane solvent mol­ecule have a fractional site occupancy of 0.25; the dichloro­methane mol­ecule is disordered about an inversion centre. The bis­(di-*o*-tolyl­phosphan­yl)methane ligand bridges an Ru—Ru bond and the monodentate phosphane ligand bonds to the third Ru atom; its S-bonded phenyl ring is disordered over two orientations in a 0.53 (4):0.47 (4) ratio. All the P atoms are equatorial with respect to the Ru_3_ triangle: each Ru atom also bears one equatorial and two axial terminal carbonyl ligands. The dihedral angles between the two benzene rings attached to each P atom of the diphenyl­phosphanyl ligand are 68.4 (2) and 71.5 (2)°. In the crystal, mol­ecules are linked into [001] chains *via* inter­molecular C—H⋯O hydrogen bonds. Weak inter­molecular C—H⋯π inter­actions also occur.

## Related literature

For general background to *triangulo*-triruthenium derivatives, see: Bruce *et al.* (1985 ▶, 1988*a* ▶,*b* ▶). For related structures, see: Shawkataly *et al.* (1998 ▶, 2004 ▶, 2010 ▶). For the synthesis of diphen­yl((phenyl­thio)­meth­yl)phosphine, see: Sanger (1983 ▶) and for that of bis­(di-*o*-tolyl­phosphan­yl)methane, see: Filby *et al.* (2006 ▶). For the stability of the temperature controller used in the data collection, see: Cosier & Glazer (1986 ▶).
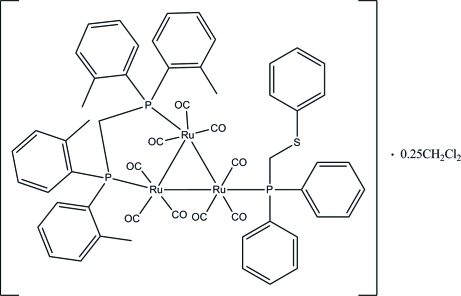


## Experimental

### 

#### Crystal data

[Ru_3_(C_29_H_30_P_2_)(C_19_H_17_PS)(CO)_9_]·0.25CH_2_Cl_2_
*M* *_r_* = 1325.36
Monoclinic, 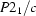

*a* = 11.022 (2) Å
*b* = 28.576 (6) Å
*c* = 18.454 (4) Å
β = 106.069 (3)°
*V* = 5585 (2) Å^3^
*Z* = 4
Mo *K*α radiation
μ = 1.00 mm^−1^
*T* = 100 K
0.15 × 0.09 × 0.08 mm

#### Data collection

Bruker APEXII DUO CCD diffractometer
Absorption correction: multi-scan (*SADABS*; Bruker, 2009 ▶) *T*_min_ = 0.862, *T*_max_ = 0.928
39779 measured reflections
12798 independent reflections
8500 reflections with *I* > 2σ(*I*)
*R*_int_ = 0.066

#### Refinement

*R*[*F* ^2^ > 2σ(*F* ^2^)] = 0.040
*wR*(*F* ^2^) = 0.092
*S* = 1.02
12798 reflections
699 parameters
180 restraints
H-atom parameters constrained
Δρ_max_ = 0.55 e Å^−3^
Δρ_min_ = −0.66 e Å^−3^

### 

Data collection: *APEX2* (Bruker, 2009 ▶); cell refinement: *SAINT* (Bruker, 2009 ▶); data reduction: *SAINT*; program(s) used to solve structure: *SHELXTL* (Sheldrick, 2008 ▶); program(s) used to refine structure: *SHELXTL*; molecular graphics: *SHELXTL*; software used to prepare material for publication: *SHELXTL* and *PLATON* (Spek, 2009 ▶).

## Supplementary Material

Crystal structure: contains datablocks global, I. DOI: 10.1107/S160053681100081X/hb5785sup1.cif

Structure factors: contains datablocks I. DOI: 10.1107/S160053681100081X/hb5785Isup2.hkl

Additional supplementary materials:  crystallographic information; 3D view; checkCIF report

## Figures and Tables

**Table 1:** Selected bond lengths (Å)

Ru1—P1	2.3594 (11)
Ru2—P2	2.3476 (11)
Ru3—P3	2.3289 (12)

**Table 2:** Hydrogen-bond geometry (Å, °) *Cg*1 and *Cg*2 are the centroids of the C7–C12 and C14–C19 benzene rings, respectively.

*D*—H⋯*A*	*D*—H	H⋯*A*	*D*⋯*A*	*D*—H⋯*A*
C9—H9*A*⋯O1^i^	0.93	2.53	3.330 (5)	144
C29—H29*B*⋯*Cg*1^ii^	0.96	2.97	3.554 (5)	121
C40—H40*A*⋯*Cg*1^iii^	0.93	2.92	3.670 (6)	139
C58—H58*A*⋯*Cg*2^iv^	0.97	2.67	3.585 (19)	158

Symmetry codes: (i) 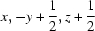; (ii) 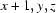; (iii) 
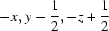
; (iv) 
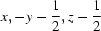
.

**Fig. 1.**
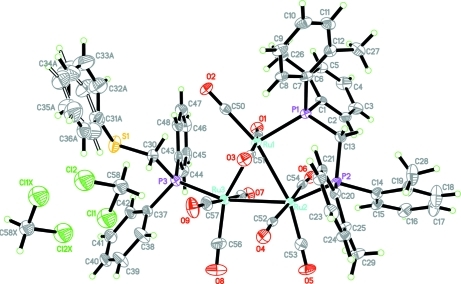
The molecular structure of the title compound with 50% probability ellipsoids for non-H atoms. Minor disorder component is shown with open bonds. Atoms with suffix X are generated by the symmetry operation (-x, -y, 1 - z).

**Fig. 2.**
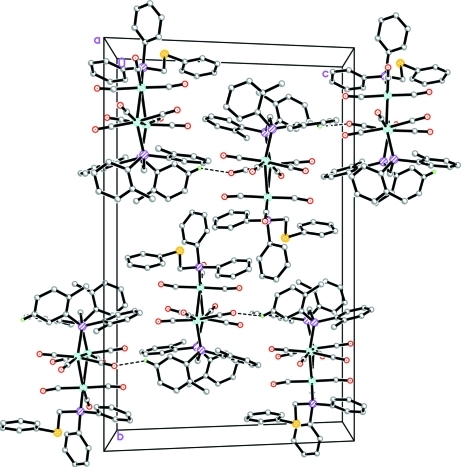
The packing of the title compound, viewed down the a axis, showing the molecules linked into chains along c axis. Hydrogen atoms not involved in the hydrogen-bonding (dashed lines), minor disorder component and solvent molecules have been omitted for clarity.

## supplementary crystallographic information

### Comment

A large number of substituted derivatives, Ru_3_(CO)_12-n_*L*_n_ (*L* = group 15 ligand) have been reported (Bruce *et al.*, 1985, 1988*a*,*b*). As part of our study on the substitution of transition metal-carbonyl clusters with mixed-ligand complexes, we have published several structures of *triangulo*-triruthenium-carbonyl clusters containing mixed P/As and P/Sb ligands (Shawkataly *et al.*, 1998, 2004, 2010). Herein we report the synthesis and structure of the title compound.

The asymmetric unit of title *triangulo*-triruthenium compound consists of one *triangulo*-triruthenium complex molecule and one-quarter molecule of dichloromethane solvent. The dichloromethane solvent lies across a crystallographic inversion center leading to the disorder of this solvent molecule over two positions. The bis(di-*o*-tolylphosphanyl)methane ligand bridges the Ru1–Ru2 bond and the monodentate phosphine ligand bonds to the Ru3 atom. Both phosphine ligands are equatorial with respect to the Ru_3_ triangle. Additionally, each Ru atom carries one equatorial and two axial terminal carbonyl ligands (Fig 1). The dihedral angles between the two benzene rings (C1–C6/C7–C12 and C14–C19/C20–C25) are 68.4 (2) and 71.5 (2)° for the two diphenylphosphanyl groups respectively.

In the crystal, the molecules are linked into one-dimensional chains along *c* axis *via* intermolecular C9—H9A···O1 hydrogen bonds (Fig. 2, Table 1). Weak intermolecular C—H···π (Table 1) interactions stabilize the crystal structure.

### Experimental

All manipulations were performed under a dry oxygen-free nitrogen atmosphere using standard Schlenk techniques. All solvents were dried over sodium and distilled from sodium benzophenone ketyl under dry oxygen free nitrogen. Diphenyl((phenylthio)methyl)phosphine (Sanger, 1983) and bis(di-*o*-tolylphosphanyl)methane (Filby *et al.*, 2006) were prepared by the reported procedures. Ru_3_(CO)_10_(µ-(2-CH_3_C_6_H_4_)_2_PCH_2_P(2-CH_3_C_6_H_4_)_2_) was prepared by reacting Ru_3_(CO)_12_ with bis(di-*o*-tolylphosphanyl)methane in presence of sodium benzophenone ketyl radical in THF. The title compound was obtained by refluxing equimolar quantities of Ru_3_(CO)_10_(µ-(2-(CH_3_C_6_H_4_)_2_PCH_2_P(2-CH_3_C_6_H_4_)_2_) and P(CH_2_SC_6_H_5_)(C_6_H_5_)_2_ in hexane under nitrogen atmosphere. Orange blocks of the title compound were grown by slow solvent / solvent diffusion of CH_3_OH into CH_2_Cl_2_.

### Refinement

All hydrogen atoms were positioned geometrically and refined using a riding model with C—H = 0.93–0.97 Å and *U*_iso_(H) = 1.2 or 1.5*U*_eq_(C). The C31–C36 benzene ring is disordered over two positions with refined site occupancies of 0.53 (4):0.47 (4). The disordered components are subjected to simulation restrain. The dichloromethane molecule is refined isotropically and with fixed occupancy of 0.25. The maximum and minimum residual electron density peaks of 0.55 and -0.66 e Å^-3^ were located 0.70 and 0.68 Å from the Cl1 and Ru2 atom, respectively.

### Crystal data

**Table:**

[Ru_3_(C_29_H_30_P_2_)(C_19_H_17_PS)(CO)_9_]·0.25CH_2_Cl_2_	*F*(000) = 2658
*M**_r_* = 1325.36	*D*_x_ = 1.576 Mg m^−^^3^
Monoclinic, *P*2_1_/*c*	Mo *K*α radiation, λ = 0.71073 Å
Hall symbol: -P 2ybc	Cell parameters from 5023 reflections
*a* = 11.022 (2) Å	θ = 2.4–27.9°
*b* = 28.576 (6) Å	µ = 1.00 mm^−^^1^
*c* = 18.454 (4) Å	*T* = 100 K
β = 106.069 (3)°	Block, orange
*V* = 5585 (2) Å^3^	0.15 × 0.09 × 0.08 mm
*Z* = 4	

### Data collection

**Table:**

Bruker APEXII DUO CCD diffractometer	12798 independent reflections
Radiation source: fine-focus sealed tube	8500 reflections with *I* > 2σ(*I*)
graphite	*R*_int_ = 0.066
φ and ω scans	θ_max_ = 27.5°, θ_min_ = 2.4°
Absorption correction: multi-scan (*SADABS*; Bruker, 2009)	*h* = −12→14
*T*_min_ = 0.862, *T*_max_ = 0.928	*k* = −35→37
39779 measured reflections	*l* = −23→23

### Refinement

**Table:**

Refinement on *F*^2^	Primary atom site location: structure-invariant direct methods
Least-squares matrix: full	Secondary atom site location: difference Fourier map
*R*[*F*^2^ > 2σ(*F*^2^)] = 0.040	Hydrogen site location: inferred from neighbouring sites
*wR*(*F*^2^) = 0.092	H-atom parameters constrained
*S* = 1.02	*w* = 1/[σ^2^(*F*_o_^2^) + (0.0285*P*)^2^ + 2.1069*P*] where *P* = (*F*_o_^2^ + 2*F*_c_^2^)/3
12798 reflections	(Δ/σ)_max_ = 0.001
699 parameters	Δρ_max_ = 0.55 e Å^−^^3^
180 restraints	Δρ_min_ = −0.66 e Å^−^^3^

### Special details

**Table:**

Experimental. The crystal was placed in the cold stream of an Oxford Cryosystems Cobra open-flow nitrogen cryostat (Cosier & Glazer, 1986) operating at 100.0 (1) K.
Geometry. All e.s.d.'s (except the e.s.d. in the dihedral angle between two l.s. planes) are estimated using the full covariance matrix. The cell e.s.d.'s are taken into account individually in the estimation of e.s.d.'s in distances, angles and torsion angles; correlations between e.s.d.'s in cell parameters are only used when they are defined by crystal symmetry. An approximate (isotropic) treatment of cell e.s.d.'s is used for estimating e.s.d.'s involving l.s. planes.
Refinement. Refinement of *F*^2^ against ALL reflections. The weighted *R*-factor *wR* and goodness of fit *S* are based on *F*^2^, conventional *R*-factors *R* are based on *F*, with *F* set to zero for negative *F*^2^. The threshold expression of *F*^2^ > σ(*F*^2^) is used only for calculating *R*-factors(gt) *etc*. and is not relevant to the choice of reflections for refinement. *R*-factors based on *F*^2^ are statistically about twice as large as those based on *F*, and *R*- factors based on ALL data will be even larger.

### Fractional atomic coordinates and isotropic or equivalent isotropic displacement parameters (Å^2^)

**Table:**

	*x*	*y*	*z*	*U*_iso_*/*U*_eq_	Occ. (<1)
Ru1	−0.14245 (3)	0.203379 (11)	0.156805 (15)	0.01570 (8)	
Ru2	0.09910 (3)	0.204897 (11)	0.129353 (15)	0.01651 (8)	
Ru3	−0.01551 (3)	0.117561 (11)	0.141835 (17)	0.01934 (8)	
S1	−0.34596 (12)	0.01710 (4)	0.22273 (7)	0.0368 (3)	
P1	−0.16719 (9)	0.28466 (4)	0.13530 (5)	0.0168 (2)	
P2	0.12747 (9)	0.28484 (3)	0.15843 (5)	0.0169 (2)	
P3	−0.16771 (10)	0.06123 (4)	0.14285 (6)	0.0209 (2)	
O1	−0.3047 (3)	0.18309 (10)	−0.00412 (14)	0.0258 (6)	
O2	−0.3753 (3)	0.17910 (10)	0.20596 (15)	0.0274 (7)	
O3	0.0073 (2)	0.20921 (10)	0.32350 (14)	0.0266 (7)	
O4	0.2607 (3)	0.17983 (10)	0.28865 (14)	0.0288 (7)	
O5	0.3265 (3)	0.17506 (11)	0.07823 (16)	0.0358 (8)	
O6	−0.0517 (2)	0.22118 (10)	−0.03527 (14)	0.0251 (6)	
O7	−0.1001 (3)	0.11920 (10)	−0.03116 (15)	0.0311 (7)	
O8	0.2027 (3)	0.05687 (14)	0.1291 (2)	0.0571 (10)	
O9	0.0685 (3)	0.10898 (10)	0.31393 (16)	0.0409 (8)	
C1	−0.2515 (3)	0.29940 (13)	0.03786 (19)	0.0174 (8)	
C2	−0.1848 (4)	0.31381 (14)	−0.0127 (2)	0.0243 (9)	
H2A	−0.0973	0.3161	0.0038	0.029*	
C3	−0.2464 (4)	0.32457 (16)	−0.0863 (2)	0.0330 (11)	
H3A	−0.2002	0.3338	−0.1191	0.040*	
C4	−0.3756 (4)	0.32185 (16)	−0.1116 (2)	0.0349 (11)	
H4A	−0.4174	0.3302	−0.1609	0.042*	
C5	−0.4422 (4)	0.30662 (14)	−0.0634 (2)	0.0282 (10)	
H5A	−0.5296	0.3044	−0.0811	0.034*	
C6	−0.3832 (3)	0.29441 (14)	0.0110 (2)	0.0203 (8)	
C7	−0.2427 (3)	0.31845 (14)	0.1965 (2)	0.0194 (8)	
C8	−0.2342 (3)	0.29804 (14)	0.2660 (2)	0.0213 (9)	
H8A	−0.1986	0.2684	0.2761	0.026*	
C9	−0.2771 (4)	0.32042 (15)	0.3208 (2)	0.0251 (9)	
H9A	−0.2676	0.3065	0.3676	0.030*	
C10	−0.3339 (4)	0.36350 (16)	0.3049 (2)	0.0323 (11)	
H10A	−0.3658	0.3785	0.3405	0.039*	
C11	−0.3436 (4)	0.38439 (15)	0.2366 (2)	0.0293 (10)	
H11A	−0.3829	0.4134	0.2267	0.035*	
C12	−0.2963 (4)	0.36350 (14)	0.1813 (2)	0.0244 (9)	
C13	−0.0186 (3)	0.31861 (13)	0.1525 (2)	0.0230 (9)	
H13A	−0.0288	0.3414	0.1123	0.028*	
H13B	−0.0068	0.3358	0.1993	0.028*	
C14	0.1976 (4)	0.32245 (14)	0.0999 (2)	0.0233 (9)	
C15	0.1957 (3)	0.30541 (15)	0.0289 (2)	0.0229 (9)	
H15A	0.1674	0.2750	0.0161	0.028*	
C16	0.2342 (4)	0.33195 (16)	−0.0233 (2)	0.0319 (11)	
H16A	0.2295	0.3201	−0.0709	0.038*	
C17	0.2792 (5)	0.37592 (18)	−0.0034 (3)	0.0474 (14)	
H17A	0.3068	0.3941	−0.0375	0.057*	
C18	0.2841 (5)	0.39359 (18)	0.0663 (3)	0.0490 (14)	
H18A	0.3168	0.4234	0.0786	0.059*	
C19	0.2417 (4)	0.36852 (15)	0.1195 (2)	0.0319 (10)	
C20	0.2198 (3)	0.29290 (13)	0.25651 (19)	0.0175 (8)	
C21	0.1587 (4)	0.29948 (13)	0.3124 (2)	0.0203 (8)	
H21A	0.0710	0.3009	0.2989	0.024*	
C22	0.2255 (4)	0.30390 (13)	0.3873 (2)	0.0242 (9)	
H22A	0.1831	0.3081	0.4239	0.029*	
C23	0.3561 (4)	0.30198 (14)	0.4075 (2)	0.0260 (10)	
H23A	0.4022	0.3064	0.4574	0.031*	
C24	0.4167 (4)	0.29364 (14)	0.3536 (2)	0.0233 (9)	
H24A	0.5044	0.2917	0.3680	0.028*	
C25	0.3519 (3)	0.28793 (13)	0.2774 (2)	0.0198 (8)	
C26	−0.4634 (4)	0.27819 (15)	0.0604 (2)	0.0263 (10)	
H26A	−0.5421	0.2662	0.0294	0.039*	
H26B	−0.4794	0.3040	0.0897	0.039*	
H26C	−0.4198	0.2540	0.0936	0.039*	
C27	−0.3046 (4)	0.39113 (15)	0.1106 (2)	0.0313 (10)	
H27A	−0.3243	0.4231	0.1184	0.047*	
H27B	−0.3697	0.3782	0.0697	0.047*	
H27C	−0.2252	0.3897	0.0988	0.047*	
C28	0.2465 (5)	0.39181 (16)	0.1941 (3)	0.0437 (13)	
H28A	0.2626	0.4247	0.1909	0.065*	
H28B	0.3128	0.3780	0.2334	0.065*	
H28C	0.1672	0.3875	0.2053	0.065*	
C29	0.4268 (4)	0.27706 (16)	0.2230 (2)	0.0300 (10)	
H29A	0.5022	0.2605	0.2488	0.045*	
H29B	0.4491	0.3057	0.2027	0.045*	
H29C	0.3772	0.2580	0.1828	0.045*	
C30	−0.2357 (4)	0.06438 (14)	0.2232 (2)	0.0249 (9)	
H30A	−0.1680	0.0632	0.2698	0.030*	
H30B	−0.2789	0.0941	0.2218	0.030*	
C31A	−0.3953 (4)	0.03194 (16)	0.3037 (2)	0.030 (5)	0.53 (4)
C32A	−0.5173 (9)	0.0494 (7)	0.2898 (3)	0.052 (4)	0.53 (4)
H32A	−0.5660	0.0542	0.2405	0.063*	0.53 (4)
C33A	−0.5664 (6)	0.0597 (9)	0.3497 (5)	0.065 (4)	0.53 (4)
H33A	−0.6480	0.0714	0.3405	0.078*	0.53 (4)
C34A	−0.4935 (6)	0.0526 (5)	0.4234 (4)	0.063 (4)	0.53 (4)
H34A	−0.5264	0.0595	0.4635	0.076*	0.53 (4)
C35A	−0.3715 (10)	0.03513 (18)	0.43726 (15)	0.046 (4)	0.53 (4)
H35A	−0.3228	0.0303	0.4866	0.056*	0.53 (4)
C36A	−0.3224 (7)	0.0248 (3)	0.3774 (3)	0.035 (3)	0.53 (4)
H36A	−0.2408	0.0131	0.3866	0.042*	0.53 (4)
C31B	−0.4008 (4)	0.02967 (18)	0.30122 (19)	0.033 (6)	0.47 (4)
C32B	−0.5287 (3)	0.0312 (7)	0.2968 (4)	0.058 (4)	0.47 (4)
H32B	−0.5885	0.0287	0.2502	0.069*	0.47 (4)
C33B	−0.5671 (6)	0.0365 (9)	0.3620 (6)	0.065 (4)	0.47 (4)
H33B	−0.6527	0.0375	0.3590	0.078*	0.47 (4)
C34B	−0.4777 (10)	0.0402 (5)	0.4316 (4)	0.058 (4)	0.47 (4)
H34B	−0.5035	0.0437	0.4752	0.070*	0.47 (4)
C35B	−0.3498 (9)	0.0387 (3)	0.43603 (19)	0.060 (5)	0.47 (4)
H35B	−0.2900	0.0412	0.4826	0.072*	0.47 (4)
C36B	−0.3114 (4)	0.0334 (4)	0.3708 (4)	0.053 (4)	0.47 (4)
H36B	−0.2258	0.0324	0.3738	0.063*	0.47 (4)
C37	−0.1059 (4)	0.00157 (14)	0.1538 (2)	0.0248 (9)	
C38	0.0052 (5)	−0.00675 (16)	0.2102 (3)	0.0411 (12)	
H38A	0.0448	0.0180	0.2403	0.049*	
C39	0.0580 (5)	−0.05091 (16)	0.2224 (3)	0.0457 (13)	
H39A	0.1307	−0.0559	0.2616	0.055*	
C40	0.0030 (5)	−0.08741 (16)	0.1765 (3)	0.0384 (12)	
H40A	0.0404	−0.1169	0.1827	0.046*	
C41	−0.1074 (5)	−0.07998 (16)	0.1217 (3)	0.0382 (11)	
H41A	−0.1461	−0.1049	0.0917	0.046*	
C42	−0.1626 (4)	−0.03638 (14)	0.1100 (2)	0.0311 (10)	
H42A	−0.2382	−0.0323	0.0726	0.037*	
C43	−0.3036 (4)	0.06080 (13)	0.0597 (2)	0.0221 (9)	
C44	−0.2919 (4)	0.04285 (14)	−0.0075 (2)	0.0280 (10)	
H44A	−0.2155	0.0300	−0.0097	0.034*	
C45	−0.3939 (4)	0.04392 (15)	−0.0721 (2)	0.0336 (11)	
H45A	−0.3865	0.0304	−0.1165	0.040*	
C46	−0.5057 (4)	0.06497 (15)	−0.0703 (2)	0.0347 (11)	
H46A	−0.5732	0.0663	−0.1137	0.042*	
C47	−0.5163 (4)	0.08400 (15)	−0.0035 (3)	0.0346 (11)	
H47A	−0.5913	0.0984	−0.0021	0.041*	
C48	−0.4170 (4)	0.08181 (14)	0.0613 (2)	0.0276 (10)	
H48A	−0.4258	0.0944	0.1061	0.033*	
C49	−0.2362 (3)	0.18993 (13)	0.0545 (2)	0.0190 (8)	
C50	−0.2871 (4)	0.18831 (13)	0.1871 (2)	0.0205 (9)	
C51	−0.0414 (3)	0.20681 (14)	0.2606 (2)	0.0198 (8)	
C52	0.1944 (3)	0.18872 (13)	0.2310 (2)	0.0200 (8)	
C53	0.2411 (4)	0.18684 (15)	0.0974 (2)	0.0250 (9)	
C54	−0.0023 (3)	0.21497 (13)	0.0274 (2)	0.0199 (8)	
C55	−0.0706 (4)	0.12134 (14)	0.0332 (2)	0.0238 (9)	
C56	0.1201 (4)	0.07923 (17)	0.1345 (3)	0.0364 (11)	
C57	0.0378 (4)	0.11576 (14)	0.2504 (2)	0.0287 (10)	
Cl1	0.0339 (6)	0.0158 (2)	0.4242 (3)	0.0608 (15)*	0.25
Cl2	−0.0173 (6)	0.0494 (2)	0.5618 (3)	0.0599 (15)*	0.25
C58	−0.0061 (18)	0.0667 (6)	0.4726 (10)	0.036 (4)*	0.25
H58A	0.0585	0.0905	0.4781	0.043*	0.25
H58B	−0.0859	0.0798	0.4434	0.043*	0.25

### Atomic displacement parameters (Å^2^)

**Table:**

	*U*^11^	*U*^22^	*U*^33^	*U*^12^	*U*^13^	*U*^23^
Ru1	0.01344 (15)	0.02026 (17)	0.01309 (14)	0.00001 (13)	0.00318 (11)	0.00101 (12)
Ru2	0.01358 (16)	0.02206 (18)	0.01362 (14)	−0.00093 (13)	0.00329 (11)	−0.00147 (12)
Ru3	0.01813 (16)	0.01951 (17)	0.01903 (15)	0.00044 (14)	0.00291 (11)	−0.00097 (13)
S1	0.0514 (8)	0.0312 (7)	0.0345 (6)	−0.0142 (6)	0.0228 (5)	−0.0077 (5)
P1	0.0152 (5)	0.0225 (6)	0.0118 (4)	0.0006 (4)	0.0023 (4)	−0.0001 (4)
P2	0.0138 (5)	0.0219 (6)	0.0145 (4)	−0.0024 (4)	0.0033 (4)	−0.0008 (4)
P3	0.0235 (5)	0.0171 (5)	0.0210 (5)	0.0005 (4)	0.0042 (4)	0.0006 (4)
O1	0.0249 (15)	0.0328 (17)	0.0192 (14)	−0.0014 (13)	0.0049 (12)	−0.0026 (12)
O2	0.0223 (16)	0.0363 (18)	0.0274 (15)	−0.0035 (13)	0.0132 (12)	0.0007 (13)
O3	0.0235 (15)	0.0366 (18)	0.0164 (13)	0.0010 (13)	−0.0001 (11)	0.0018 (12)
O4	0.0269 (16)	0.0392 (18)	0.0165 (14)	−0.0008 (14)	−0.0004 (12)	0.0041 (12)
O5	0.0248 (17)	0.057 (2)	0.0290 (17)	0.0106 (16)	0.0135 (13)	0.0004 (15)
O6	0.0221 (15)	0.0332 (17)	0.0177 (14)	−0.0031 (13)	0.0018 (11)	0.0004 (12)
O7	0.0423 (18)	0.0302 (17)	0.0206 (15)	−0.0017 (14)	0.0085 (13)	−0.0015 (12)
O8	0.035 (2)	0.069 (3)	0.068 (3)	0.0206 (19)	0.0147 (18)	−0.007 (2)
O9	0.059 (2)	0.0323 (19)	0.0230 (16)	−0.0101 (16)	−0.0034 (14)	0.0057 (13)
C1	0.0186 (19)	0.021 (2)	0.0121 (16)	0.0061 (16)	0.0033 (14)	0.0006 (15)
C2	0.019 (2)	0.029 (2)	0.025 (2)	0.0048 (18)	0.0072 (16)	0.0049 (17)
C3	0.040 (3)	0.043 (3)	0.020 (2)	0.012 (2)	0.0157 (19)	0.0071 (19)
C4	0.035 (3)	0.050 (3)	0.017 (2)	0.009 (2)	0.0019 (18)	0.0042 (19)
C5	0.024 (2)	0.035 (3)	0.022 (2)	0.0042 (19)	−0.0001 (16)	−0.0031 (18)
C6	0.020 (2)	0.025 (2)	0.0153 (17)	0.0034 (17)	0.0031 (14)	−0.0018 (16)
C7	0.0123 (19)	0.028 (2)	0.0167 (18)	−0.0023 (16)	0.0019 (14)	−0.0076 (16)
C8	0.0134 (19)	0.029 (2)	0.0196 (18)	0.0023 (17)	0.0011 (14)	−0.0019 (17)
C9	0.019 (2)	0.040 (3)	0.0171 (19)	−0.0014 (19)	0.0052 (15)	−0.0060 (17)
C10	0.027 (2)	0.039 (3)	0.034 (2)	−0.003 (2)	0.0134 (19)	−0.018 (2)
C11	0.030 (2)	0.024 (2)	0.033 (2)	0.0019 (19)	0.0078 (18)	−0.0102 (18)
C12	0.021 (2)	0.028 (2)	0.023 (2)	−0.0015 (18)	0.0033 (16)	−0.0019 (17)
C13	0.020 (2)	0.020 (2)	0.027 (2)	0.0002 (18)	0.0036 (16)	0.0004 (17)
C14	0.020 (2)	0.029 (2)	0.0199 (19)	−0.0032 (18)	0.0032 (16)	0.0020 (16)
C15	0.0145 (19)	0.033 (2)	0.0196 (19)	−0.0008 (17)	0.0018 (15)	0.0032 (17)
C16	0.029 (2)	0.048 (3)	0.018 (2)	−0.003 (2)	0.0052 (17)	0.0052 (19)
C17	0.060 (3)	0.047 (3)	0.036 (3)	−0.016 (3)	0.014 (2)	0.015 (2)
C18	0.072 (4)	0.038 (3)	0.040 (3)	−0.023 (3)	0.020 (3)	−0.002 (2)
C19	0.038 (3)	0.034 (3)	0.026 (2)	−0.009 (2)	0.0133 (19)	0.0052 (19)
C20	0.0179 (19)	0.019 (2)	0.0150 (17)	−0.0022 (16)	0.0030 (14)	−0.0026 (15)
C21	0.021 (2)	0.016 (2)	0.026 (2)	−0.0014 (17)	0.0104 (16)	0.0000 (16)
C22	0.031 (2)	0.026 (2)	0.0181 (19)	0.0000 (18)	0.0112 (16)	−0.0006 (16)
C23	0.033 (2)	0.026 (2)	0.0156 (18)	0.0006 (19)	0.0007 (16)	−0.0021 (16)
C24	0.0154 (19)	0.032 (2)	0.0199 (19)	0.0010 (18)	0.0002 (15)	0.0020 (17)
C25	0.0150 (19)	0.025 (2)	0.0201 (19)	−0.0008 (17)	0.0055 (15)	−0.0021 (16)
C26	0.017 (2)	0.034 (2)	0.023 (2)	0.0016 (18)	−0.0028 (16)	0.0007 (17)
C27	0.039 (3)	0.027 (2)	0.026 (2)	0.008 (2)	0.0068 (18)	0.0005 (18)
C28	0.065 (4)	0.030 (3)	0.038 (3)	−0.020 (2)	0.017 (2)	−0.005 (2)
C29	0.016 (2)	0.047 (3)	0.026 (2)	−0.0009 (19)	0.0045 (17)	−0.0130 (19)
C30	0.028 (2)	0.023 (2)	0.024 (2)	−0.0024 (18)	0.0072 (17)	−0.0001 (16)
C31A	0.036 (9)	0.029 (9)	0.033 (9)	−0.003 (8)	0.025 (8)	0.006 (8)
C32A	0.040 (7)	0.073 (9)	0.040 (6)	0.007 (6)	0.006 (5)	0.008 (5)
C33A	0.045 (7)	0.102 (11)	0.052 (7)	0.019 (7)	0.020 (5)	0.009 (7)
C34A	0.073 (9)	0.082 (9)	0.040 (7)	0.030 (7)	0.025 (6)	−0.004 (6)
C35A	0.056 (7)	0.059 (9)	0.025 (7)	0.010 (7)	0.013 (5)	−0.014 (6)
C36A	0.042 (7)	0.035 (6)	0.030 (6)	−0.002 (5)	0.013 (5)	0.000 (5)
C31B	0.039 (11)	0.029 (10)	0.026 (10)	0.002 (9)	0.003 (9)	−0.009 (9)
C32B	0.051 (8)	0.079 (9)	0.051 (7)	−0.009 (7)	0.028 (6)	−0.005 (7)
C33B	0.056 (8)	0.082 (12)	0.072 (9)	0.006 (7)	0.042 (7)	−0.001 (8)
C34B	0.075 (10)	0.064 (8)	0.047 (8)	0.005 (8)	0.038 (7)	−0.001 (6)
C35B	0.068 (9)	0.070 (11)	0.045 (10)	0.000 (9)	0.020 (8)	0.007 (9)
C36B	0.056 (9)	0.073 (9)	0.030 (8)	0.014 (8)	0.013 (7)	0.003 (7)
C37	0.028 (2)	0.023 (2)	0.025 (2)	0.0029 (18)	0.0099 (17)	0.0044 (17)
C38	0.047 (3)	0.027 (3)	0.040 (3)	0.008 (2)	−0.004 (2)	0.000 (2)
C39	0.050 (3)	0.032 (3)	0.048 (3)	0.014 (2)	0.003 (2)	0.010 (2)
C40	0.048 (3)	0.021 (3)	0.053 (3)	0.011 (2)	0.027 (2)	0.010 (2)
C41	0.046 (3)	0.023 (3)	0.050 (3)	0.000 (2)	0.020 (2)	−0.005 (2)
C42	0.030 (2)	0.018 (2)	0.042 (3)	−0.0025 (19)	0.0059 (19)	−0.0002 (18)
C43	0.025 (2)	0.017 (2)	0.022 (2)	−0.0077 (17)	0.0035 (16)	0.0021 (16)
C44	0.029 (2)	0.028 (2)	0.028 (2)	−0.0056 (19)	0.0115 (18)	−0.0013 (18)
C45	0.043 (3)	0.030 (3)	0.026 (2)	−0.014 (2)	0.0062 (19)	−0.0026 (18)
C46	0.033 (3)	0.033 (3)	0.029 (2)	−0.010 (2)	−0.0049 (19)	0.0021 (19)
C47	0.026 (2)	0.031 (3)	0.042 (3)	−0.001 (2)	0.0004 (19)	0.002 (2)
C48	0.031 (2)	0.020 (2)	0.032 (2)	−0.0008 (19)	0.0085 (18)	−0.0002 (17)
C49	0.018 (2)	0.023 (2)	0.0189 (19)	0.0011 (16)	0.0100 (15)	0.0002 (15)
C50	0.023 (2)	0.021 (2)	0.0158 (18)	0.0036 (17)	0.0031 (15)	0.0013 (15)
C51	0.0129 (18)	0.024 (2)	0.023 (2)	0.0010 (17)	0.0071 (15)	0.0012 (16)
C52	0.016 (2)	0.022 (2)	0.025 (2)	0.0003 (16)	0.0101 (16)	0.0003 (16)
C53	0.026 (2)	0.030 (2)	0.0169 (19)	−0.0007 (19)	0.0018 (16)	0.0006 (16)
C54	0.0134 (19)	0.022 (2)	0.025 (2)	−0.0037 (16)	0.0068 (15)	−0.0039 (16)
C55	0.023 (2)	0.018 (2)	0.031 (2)	0.0005 (17)	0.0091 (17)	−0.0009 (17)
C56	0.024 (2)	0.042 (3)	0.042 (3)	0.004 (2)	0.007 (2)	−0.001 (2)
C57	0.032 (2)	0.018 (2)	0.034 (2)	−0.0035 (19)	0.0054 (19)	−0.0019 (18)

### Geometric parameters (Å, °)

**Table:**

Ru1—C50	1.880 (4)	C21—C22	1.381 (5)
Ru1—C49	1.924 (4)	C21—H21A	0.9300
Ru1—C51	1.933 (4)	C22—C23	1.384 (5)
Ru1—P1	2.3594 (11)	C22—H22A	0.9300
Ru1—Ru2	2.8449 (7)	C23—C24	1.364 (6)
Ru1—Ru3	2.8744 (6)	C23—H23A	0.9300
Ru2—C53	1.891 (5)	C24—C25	1.399 (5)
Ru2—C54	1.925 (4)	C24—H24A	0.9300
Ru2—C52	1.936 (4)	C25—C29	1.498 (5)
Ru2—P2	2.3476 (11)	C26—H26A	0.9600
Ru2—Ru3	2.8352 (6)	C26—H26B	0.9600
Ru3—C56	1.888 (5)	C26—H26C	0.9600
Ru3—C57	1.926 (4)	C27—H27A	0.9600
Ru3—C55	1.931 (4)	C27—H27B	0.9600
Ru3—P3	2.3289 (12)	C27—H27C	0.9600
S1—C31B	1.754 (3)	C28—H28A	0.9600
S1—C31A	1.777 (3)	C28—H28B	0.9600
S1—C30	1.816 (4)	C28—H28C	0.9600
P1—C1	1.830 (3)	C29—H29A	0.9600
P1—C7	1.849 (4)	C29—H29B	0.9600
P1—C13	1.854 (4)	C29—H29C	0.9600
P2—C20	1.829 (3)	C30—H30A	0.9700
P2—C14	1.839 (4)	C30—H30B	0.9700
P2—C13	1.854 (4)	C31A—C32A	1.3900
P3—C43	1.824 (4)	C31A—C36A	1.3900
P3—C37	1.826 (4)	C32A—C33A	1.3900
P3—C30	1.840 (4)	C32A—H32A	0.9300
O1—C49	1.151 (4)	C33A—C34A	1.3900
O2—C50	1.149 (5)	C33A—H33A	0.9300
O3—C51	1.139 (4)	C34A—C35A	1.3900
O4—C52	1.139 (4)	C34A—H34A	0.9300
O5—C53	1.144 (5)	C35A—C36A	1.3900
O6—C54	1.148 (4)	C35A—H35A	0.9300
O7—C55	1.142 (5)	C36A—H36A	0.9300
O8—C56	1.140 (5)	C31B—C32B	1.3900
O9—C57	1.143 (5)	C31B—C36B	1.3900
C1—C2	1.401 (5)	C32B—C33B	1.3900
C1—C6	1.405 (5)	C32B—H32B	0.9300
C2—C3	1.374 (5)	C33B—C34B	1.3900
C2—H2A	0.9300	C33B—H33B	0.9300
C3—C4	1.373 (6)	C34B—C35B	1.3900
C3—H3A	0.9300	C34B—H34B	0.9300
C4—C5	1.372 (6)	C35B—C36B	1.3900
C4—H4A	0.9300	C35B—H35B	0.9300
C5—C6	1.392 (5)	C36B—H36B	0.9300
C5—H5A	0.9300	C37—C38	1.390 (6)
C6—C26	1.508 (5)	C37—C42	1.393 (5)
C7—C8	1.389 (5)	C38—C39	1.382 (6)
C7—C12	1.412 (5)	C38—H38A	0.9300
C8—C9	1.385 (5)	C39—C40	1.374 (6)
C8—H8A	0.9300	C39—H39A	0.9300
C9—C10	1.376 (6)	C40—C41	1.366 (6)
C9—H9A	0.9300	C40—H40A	0.9300
C10—C11	1.372 (6)	C41—C42	1.377 (6)
C10—H10A	0.9300	C41—H41A	0.9300
C11—C12	1.402 (6)	C42—H42A	0.9300
C11—H11A	0.9300	C43—C44	1.380 (5)
C12—C27	1.505 (5)	C43—C48	1.395 (6)
C13—H13A	0.9700	C44—C45	1.394 (6)
C13—H13B	0.9700	C44—H44A	0.9300
C14—C15	1.392 (5)	C45—C46	1.380 (6)
C14—C19	1.415 (6)	C45—H45A	0.9300
C15—C16	1.381 (5)	C46—C47	1.383 (6)
C15—H15A	0.9300	C46—H46A	0.9300
C16—C17	1.363 (6)	C47—C48	1.381 (5)
C16—H16A	0.9300	C47—H47A	0.9300
C17—C18	1.368 (7)	C48—H48A	0.9300
C17—H17A	0.9300	Cl1—C58	1.824 (19)
C18—C19	1.397 (6)	Cl1—Cl2^i^	1.896 (8)
C18—H18A	0.9300	Cl2—C58	1.755 (19)
C19—C28	1.517 (6)	Cl2—Cl1^i^	1.896 (8)
C20—C21	1.392 (5)	C58—H58A	0.9700
C20—C25	1.406 (5)	C58—H58B	0.9700
			
C50—Ru1—C49	88.68 (16)	C21—C22—C23	119.5 (4)
C50—Ru1—C51	91.17 (15)	C21—C22—H22A	120.2
C49—Ru1—C51	171.19 (16)	C23—C22—H22A	120.2
C50—Ru1—P1	101.97 (12)	C24—C23—C22	119.6 (3)
C49—Ru1—P1	91.48 (12)	C24—C23—H23A	120.2
C51—Ru1—P1	97.16 (12)	C22—C23—H23A	120.2
C50—Ru1—Ru2	165.71 (11)	C23—C24—C25	122.4 (4)
C49—Ru1—Ru2	95.92 (11)	C23—C24—H24A	118.8
C51—Ru1—Ru2	82.22 (11)	C25—C24—H24A	118.8
P1—Ru1—Ru2	91.45 (3)	C24—C25—C20	117.7 (3)
C50—Ru1—Ru3	107.98 (12)	C24—C25—C29	118.3 (3)
C49—Ru1—Ru3	83.03 (11)	C20—C25—C29	123.9 (3)
C51—Ru1—Ru3	88.64 (11)	C6—C26—H26A	109.5
P1—Ru1—Ru3	149.37 (3)	C6—C26—H26B	109.5
Ru2—Ru1—Ru3	59.434 (13)	H26A—C26—H26B	109.5
C53—Ru2—C54	92.46 (16)	C6—C26—H26C	109.5
C53—Ru2—C52	87.91 (16)	H26A—C26—H26C	109.5
C54—Ru2—C52	174.47 (16)	H26B—C26—H26C	109.5
C53—Ru2—P2	105.51 (13)	C12—C27—H27A	109.5
C54—Ru2—P2	94.73 (11)	C12—C27—H27B	109.5
C52—Ru2—P2	90.49 (12)	H27A—C27—H27B	109.5
C53—Ru2—Ru3	102.18 (13)	C12—C27—H27C	109.5
C54—Ru2—Ru3	93.42 (11)	H27A—C27—H27C	109.5
C52—Ru2—Ru3	81.11 (11)	H27B—C27—H27C	109.5
P2—Ru2—Ru3	150.71 (3)	C19—C28—H28A	109.5
C53—Ru2—Ru1	161.38 (12)	C19—C28—H28B	109.5
C54—Ru2—Ru1	81.79 (11)	H28A—C28—H28B	109.5
C52—Ru2—Ru1	96.15 (11)	C19—C28—H28C	109.5
P2—Ru2—Ru1	92.65 (3)	H28A—C28—H28C	109.5
Ru3—Ru2—Ru1	60.801 (13)	H28B—C28—H28C	109.5
C56—Ru3—C57	92.27 (19)	C25—C29—H29A	109.5
C56—Ru3—C55	89.19 (18)	C25—C29—H29B	109.5
C57—Ru3—C55	178.22 (17)	H29A—C29—H29B	109.5
C56—Ru3—P3	100.71 (15)	C25—C29—H29C	109.5
C57—Ru3—P3	89.20 (13)	H29A—C29—H29C	109.5
C55—Ru3—P3	91.53 (12)	H29B—C29—H29C	109.5
C56—Ru3—Ru2	97.21 (15)	S1—C30—P3	112.4 (2)
C57—Ru3—Ru2	95.37 (12)	S1—C30—H30A	109.1
C55—Ru3—Ru2	83.43 (12)	P3—C30—H30A	109.1
P3—Ru3—Ru2	161.31 (3)	S1—C30—H30B	109.1
C56—Ru3—Ru1	156.64 (14)	P3—C30—H30B	109.1
C57—Ru3—Ru1	86.51 (13)	H30A—C30—H30B	107.9
C55—Ru3—Ru1	91.75 (12)	C32A—C31A—C36A	120.0
P3—Ru3—Ru1	102.60 (3)	C32A—C31A—S1	115.9 (3)
Ru2—Ru3—Ru1	59.765 (17)	C36A—C31A—S1	124.0 (4)
C31B—S1—C30	103.1 (2)	C31A—C32A—C33A	120.0
C31A—S1—C30	100.3 (2)	C31A—C32A—H32A	120.0
C1—P1—C7	106.70 (17)	C33A—C32A—H32A	120.0
C1—P1—C13	103.41 (18)	C34A—C33A—C32A	120.0
C7—P1—C13	98.52 (17)	C34A—C33A—H33A	120.0
C1—P1—Ru1	113.30 (12)	C32A—C33A—H33A	120.0
C7—P1—Ru1	117.52 (13)	C35A—C34A—C33A	120.0
C13—P1—Ru1	115.52 (13)	C35A—C34A—H34A	120.0
C20—P2—C14	107.36 (17)	C33A—C34A—H34A	120.0
C20—P2—C13	103.26 (17)	C34A—C35A—C36A	120.0
C14—P2—C13	99.22 (18)	C34A—C35A—H35A	120.0
C20—P2—Ru2	110.56 (12)	C36A—C35A—H35A	120.0
C14—P2—Ru2	119.04 (14)	C35A—C36A—C31A	120.0
C13—P2—Ru2	115.75 (13)	C35A—C36A—H36A	120.0
C43—P3—C37	106.34 (18)	C31A—C36A—H36A	120.0
C43—P3—C30	104.83 (19)	C32B—C31B—C36B	120.0
C37—P3—C30	100.34 (18)	C32B—C31B—S1	122.2 (3)
C43—P3—Ru3	115.05 (13)	C36B—C31B—S1	117.4 (3)
C37—P3—Ru3	113.48 (14)	C31B—C32B—C33B	120.0
C30—P3—Ru3	115.32 (13)	C31B—C32B—H32B	120.0
C2—C1—C6	118.5 (3)	C33B—C32B—H32B	120.0
C2—C1—P1	120.3 (3)	C34B—C33B—C32B	120.0
C6—C1—P1	121.1 (3)	C34B—C33B—H33B	120.0
C3—C2—C1	121.1 (4)	C32B—C33B—H33B	120.0
C3—C2—H2A	119.4	C33B—C34B—C35B	120.0
C1—C2—H2A	119.4	C33B—C34B—H34B	120.0
C4—C3—C2	120.5 (4)	C35B—C34B—H34B	120.0
C4—C3—H3A	119.8	C34B—C35B—C36B	120.0
C2—C3—H3A	119.8	C34B—C35B—H35B	120.0
C5—C4—C3	119.2 (4)	C36B—C35B—H35B	120.0
C5—C4—H4A	120.4	C35B—C36B—C31B	120.0
C3—C4—H4A	120.4	C35B—C36B—H36B	120.0
C4—C5—C6	122.1 (4)	C31B—C36B—H36B	120.0
C4—C5—H5A	118.9	C38—C37—C42	117.5 (4)
C6—C5—H5A	118.9	C38—C37—P3	117.8 (3)
C5—C6—C1	118.5 (4)	C42—C37—P3	124.7 (3)
C5—C6—C26	118.8 (3)	C39—C38—C37	121.5 (4)
C1—C6—C26	122.6 (3)	C39—C38—H38A	119.2
C8—C7—C12	118.9 (4)	C37—C38—H38A	119.2
C8—C7—P1	114.7 (3)	C40—C39—C38	119.9 (4)
C12—C7—P1	126.1 (3)	C40—C39—H39A	120.1
C9—C8—C7	122.1 (4)	C38—C39—H39A	120.1
C9—C8—H8A	119.0	C41—C40—C39	119.3 (4)
C7—C8—H8A	119.0	C41—C40—H40A	120.4
C10—C9—C8	119.1 (4)	C39—C40—H40A	120.4
C10—C9—H9A	120.5	C40—C41—C42	121.4 (4)
C8—C9—H9A	120.5	C40—C41—H41A	119.3
C11—C10—C9	120.0 (4)	C42—C41—H41A	119.3
C11—C10—H10A	120.0	C41—C42—C37	120.3 (4)
C9—C10—H10A	120.0	C41—C42—H42A	119.8
C10—C11—C12	122.3 (4)	C37—C42—H42A	119.8
C10—C11—H11A	118.9	C44—C43—C48	119.1 (4)
C12—C11—H11A	118.9	C44—C43—P3	119.9 (3)
C11—C12—C7	117.6 (4)	C48—C43—P3	120.8 (3)
C11—C12—C27	117.5 (4)	C43—C44—C45	120.4 (4)
C7—C12—C27	124.9 (4)	C43—C44—H44A	119.8
P2—C13—P1	116.7 (2)	C45—C44—H44A	119.8
P2—C13—H13A	108.1	C46—C45—C44	120.3 (4)
P1—C13—H13A	108.1	C46—C45—H45A	119.9
P2—C13—H13B	108.1	C44—C45—H45A	119.9
P1—C13—H13B	108.1	C45—C46—C47	119.3 (4)
H13A—C13—H13B	107.3	C45—C46—H46A	120.4
C15—C14—C19	118.8 (4)	C47—C46—H46A	120.4
C15—C14—P2	116.7 (3)	C48—C47—C46	120.8 (4)
C19—C14—P2	124.3 (3)	C48—C47—H47A	119.6
C16—C15—C14	122.4 (4)	C46—C47—H47A	119.6
C16—C15—H15A	118.8	C47—C48—C43	120.1 (4)
C14—C15—H15A	118.8	C47—C48—H48A	120.0
C17—C16—C15	118.5 (4)	C43—C48—H48A	120.0
C17—C16—H16A	120.7	O1—C49—Ru1	172.0 (3)
C15—C16—H16A	120.7	O2—C50—Ru1	179.7 (4)
C16—C17—C18	120.6 (5)	O3—C51—Ru1	173.3 (3)
C16—C17—H17A	119.7	O4—C52—Ru2	173.4 (3)
C18—C17—H17A	119.7	O5—C53—Ru2	178.7 (4)
C17—C18—C19	122.5 (5)	O6—C54—Ru2	173.2 (3)
C17—C18—H18A	118.7	O7—C55—Ru3	173.5 (3)
C19—C18—H18A	118.7	O8—C56—Ru3	178.4 (5)
C18—C19—C14	117.0 (4)	O9—C57—Ru3	171.8 (4)
C18—C19—C28	118.6 (4)	C58—Cl1—Cl2^i^	132.1 (7)
C14—C19—C28	124.3 (4)	C58—Cl2—Cl1^i^	116.0 (7)
C21—C20—C25	119.2 (3)	Cl2—C58—Cl1	108.9 (10)
C21—C20—P2	119.9 (3)	Cl2—C58—H58A	109.9
C25—C20—P2	120.6 (3)	Cl1—C58—H58A	109.9
C22—C21—C20	121.3 (4)	Cl2—C58—H58B	109.9
C22—C21—H21A	119.3	Cl1—C58—H58B	109.9
C20—C21—H21A	119.3	H58A—C58—H58B	108.3
			
C50—Ru1—Ru2—C53	55.6 (6)	P1—C1—C2—C3	179.4 (3)
C49—Ru1—Ru2—C53	−52.7 (4)	C1—C2—C3—C4	0.6 (7)
C51—Ru1—Ru2—C53	118.6 (4)	C2—C3—C4—C5	−2.3 (7)
P1—Ru1—Ru2—C53	−144.3 (4)	C3—C4—C5—C6	0.9 (7)
Ru3—Ru1—Ru2—C53	25.6 (4)	C4—C5—C6—C1	2.1 (6)
C50—Ru1—Ru2—C54	128.6 (5)	C4—C5—C6—C26	179.8 (4)
C49—Ru1—Ru2—C54	20.31 (16)	C2—C1—C6—C5	−3.7 (6)
C51—Ru1—Ru2—C54	−168.36 (16)	P1—C1—C6—C5	179.4 (3)
P1—Ru1—Ru2—C54	−71.33 (12)	C2—C1—C6—C26	178.7 (4)
Ru3—Ru1—Ru2—C54	98.61 (11)	P1—C1—C6—C26	1.8 (5)
C50—Ru1—Ru2—C52	−46.3 (5)	C1—P1—C7—C8	152.3 (3)
C49—Ru1—Ru2—C52	−154.51 (16)	C13—P1—C7—C8	−100.9 (3)
C51—Ru1—Ru2—C52	16.81 (16)	Ru1—P1—C7—C8	23.8 (3)
P1—Ru1—Ru2—C52	113.84 (12)	C1—P1—C7—C12	−33.5 (4)
Ru3—Ru1—Ru2—C52	−76.22 (11)	C13—P1—C7—C12	73.4 (3)
C50—Ru1—Ru2—P2	−137.0 (5)	Ru1—P1—C7—C12	−162.0 (3)
C49—Ru1—Ru2—P2	114.71 (12)	C12—C7—C8—C9	0.2 (6)
C51—Ru1—Ru2—P2	−73.96 (12)	P1—C7—C8—C9	174.9 (3)
P1—Ru1—Ru2—P2	23.07 (3)	C7—C8—C9—C10	2.3 (6)
Ru3—Ru1—Ru2—P2	−166.99 (3)	C8—C9—C10—C11	−2.2 (6)
C50—Ru1—Ru2—Ru3	30.0 (5)	C9—C10—C11—C12	−0.5 (6)
C49—Ru1—Ru2—Ru3	−78.30 (11)	C10—C11—C12—C7	3.0 (6)
C51—Ru1—Ru2—Ru3	93.03 (12)	C10—C11—C12—C27	−176.0 (4)
P1—Ru1—Ru2—Ru3	−169.94 (3)	C8—C7—C12—C11	−2.8 (5)
C53—Ru2—Ru3—C56	12.34 (18)	P1—C7—C12—C11	−176.8 (3)
C54—Ru2—Ru3—C56	105.60 (18)	C8—C7—C12—C27	176.1 (4)
C52—Ru2—Ru3—C56	−73.55 (18)	P1—C7—C12—C27	2.1 (6)
P2—Ru2—Ru3—C56	−148.41 (15)	C20—P2—C13—P1	−114.1 (2)
Ru1—Ru2—Ru3—C56	−175.77 (14)	C14—P2—C13—P1	135.5 (2)
C53—Ru2—Ru3—C57	105.32 (17)	Ru2—P2—C13—P1	6.8 (3)
C54—Ru2—Ru3—C57	−161.42 (17)	C1—P1—C13—P2	−108.1 (2)
C52—Ru2—Ru3—C57	19.42 (17)	C7—P1—C13—P2	142.3 (2)
P2—Ru2—Ru3—C57	−55.43 (14)	Ru1—P1—C13—P2	16.2 (3)
Ru1—Ru2—Ru3—C57	−82.79 (13)	C20—P2—C14—C15	145.2 (3)
C53—Ru2—Ru3—C55	−76.00 (16)	C13—P2—C14—C15	−107.7 (3)
C54—Ru2—Ru3—C55	17.26 (16)	Ru2—P2—C14—C15	18.8 (4)
C52—Ru2—Ru3—C55	−161.90 (17)	C20—P2—C14—C19	−40.9 (4)
P2—Ru2—Ru3—C55	123.25 (13)	C13—P2—C14—C19	66.3 (4)
Ru1—Ru2—Ru3—C55	95.89 (12)	Ru2—P2—C14—C19	−167.3 (3)
C53—Ru2—Ru3—P3	−151.13 (14)	C19—C14—C15—C16	−0.6 (6)
C54—Ru2—Ru3—P3	−57.87 (15)	P2—C14—C15—C16	173.7 (3)
C52—Ru2—Ru3—P3	122.97 (15)	C14—C15—C16—C17	2.0 (6)
P2—Ru2—Ru3—P3	48.11 (11)	C15—C16—C17—C18	−1.1 (7)
Ru1—Ru2—Ru3—P3	20.76 (9)	C16—C17—C18—C19	−1.3 (9)
C53—Ru2—Ru3—Ru1	−171.89 (11)	C17—C18—C19—C14	2.7 (8)
C54—Ru2—Ru3—Ru1	−78.63 (12)	C17—C18—C19—C28	−177.7 (5)
C52—Ru2—Ru3—Ru1	102.22 (12)	C15—C14—C19—C18	−1.7 (6)
P2—Ru2—Ru3—Ru1	27.36 (5)	P2—C14—C19—C18	−175.5 (4)
C50—Ru1—Ru3—C56	−161.9 (4)	C15—C14—C19—C28	178.7 (4)
C49—Ru1—Ru3—C56	111.7 (4)	P2—C14—C19—C28	4.9 (6)
C51—Ru1—Ru3—C56	−71.1 (4)	C14—P2—C20—C21	134.1 (3)
P1—Ru1—Ru3—C56	30.7 (4)	C13—P2—C20—C21	29.9 (4)
Ru2—Ru1—Ru3—C56	10.6 (4)	Ru2—P2—C20—C21	−94.6 (3)
C50—Ru1—Ru3—C57	−74.27 (17)	C14—P2—C20—C25	−52.2 (4)
C49—Ru1—Ru3—C57	−160.60 (17)	C13—P2—C20—C25	−156.5 (3)
C51—Ru1—Ru3—C57	16.52 (17)	Ru2—P2—C20—C25	79.1 (3)
P1—Ru1—Ru3—C57	118.32 (14)	C25—C20—C21—C22	3.8 (6)
Ru2—Ru1—Ru3—C57	98.28 (13)	P2—C20—C21—C22	177.5 (3)
C50—Ru1—Ru3—C55	106.08 (16)	C20—C21—C22—C23	0.3 (6)
C49—Ru1—Ru3—C55	19.75 (17)	C21—C22—C23—C24	−3.0 (6)
C51—Ru1—Ru3—C55	−163.12 (16)	C22—C23—C24—C25	1.6 (6)
P1—Ru1—Ru3—C55	−61.32 (13)	C23—C24—C25—C20	2.5 (6)
Ru2—Ru1—Ru3—C55	−81.36 (12)	C23—C24—C25—C29	−177.7 (4)
C50—Ru1—Ru3—P3	14.13 (11)	C21—C20—C25—C24	−5.0 (6)
C49—Ru1—Ru3—P3	−72.20 (12)	P2—C20—C25—C24	−178.7 (3)
C51—Ru1—Ru3—P3	104.92 (12)	C21—C20—C25—C29	175.1 (4)
P1—Ru1—Ru3—P3	−153.28 (5)	P2—C20—C25—C29	1.4 (6)
Ru2—Ru1—Ru3—P3	−173.32 (3)	C31B—S1—C30—P3	−177.5 (2)
C50—Ru1—Ru3—Ru2	−172.55 (11)	C31A—S1—C30—P3	−178.2 (2)
C49—Ru1—Ru3—Ru2	101.12 (11)	C43—P3—C30—S1	56.0 (3)
C51—Ru1—Ru3—Ru2	−81.76 (11)	C37—P3—C30—S1	−54.1 (2)
P1—Ru1—Ru3—Ru2	20.04 (5)	Ru3—P3—C30—S1	−176.38 (14)
C50—Ru1—P1—C1	−91.35 (17)	C31B—S1—C31A—C32A	−60.50 (19)
C49—Ru1—P1—C1	−2.38 (18)	C30—S1—C31A—C32A	107.6 (9)
C51—Ru1—P1—C1	175.92 (17)	C31B—S1—C31A—C36A	116.08 (16)
Ru2—Ru1—P1—C1	93.58 (14)	C30—S1—C31A—C36A	−75.8 (8)
Ru3—Ru1—P1—C1	76.41 (15)	C36A—C31A—C32A—C33A	0.0
C50—Ru1—P1—C7	33.91 (17)	S1—C31A—C32A—C33A	176.7 (3)
C49—Ru1—P1—C7	122.88 (17)	C31A—C32A—C33A—C34A	0.0
C51—Ru1—P1—C7	−58.83 (17)	C32A—C33A—C34A—C35A	0.0
Ru2—Ru1—P1—C7	−141.16 (13)	C33A—C34A—C35A—C36A	0.0
Ru3—Ru1—P1—C7	−158.33 (12)	C34A—C35A—C36A—C31A	0.0
C50—Ru1—P1—C13	149.62 (18)	C32A—C31A—C36A—C35A	0.0
C49—Ru1—P1—C13	−121.42 (18)	S1—C31A—C36A—C35A	−176.5 (3)
C51—Ru1—P1—C13	56.88 (18)	C31A—S1—C31B—C32B	141.4 (3)
Ru2—Ru1—P1—C13	−25.46 (15)	C30—S1—C31B—C32B	129.4 (9)
Ru3—Ru1—P1—C13	−42.63 (16)	C31A—S1—C31B—C36B	−45.18 (11)
C53—Ru2—P2—C20	−87.92 (18)	C30—S1—C31B—C36B	−57.2 (8)
C54—Ru2—P2—C20	178.21 (17)	C36B—C31B—C32B—C33B	0.0
C52—Ru2—P2—C20	0.05 (17)	S1—C31B—C32B—C33B	173.3 (4)
Ru3—Ru2—P2—C20	72.55 (15)	C31B—C32B—C33B—C34B	0.0
Ru1—Ru2—P2—C20	96.23 (13)	C32B—C33B—C34B—C35B	0.0
C53—Ru2—P2—C14	37.03 (18)	C33B—C34B—C35B—C36B	0.0
C54—Ru2—P2—C14	−56.84 (18)	C34B—C35B—C36B—C31B	0.0
C52—Ru2—P2—C14	125.00 (18)	C32B—C31B—C36B—C35B	0.0
Ru3—Ru2—P2—C14	−162.50 (13)	S1—C31B—C36B—C35B	−173.6 (3)
Ru1—Ru2—P2—C14	−138.82 (14)	C43—P3—C37—C38	174.9 (4)
C53—Ru2—P2—C13	155.15 (18)	C30—P3—C37—C38	−76.2 (4)
C54—Ru2—P2—C13	61.28 (19)	Ru3—P3—C37—C38	47.4 (4)
C52—Ru2—P2—C13	−116.88 (18)	C43—P3—C37—C42	−5.6 (4)
Ru3—Ru2—P2—C13	−44.38 (16)	C30—P3—C37—C42	103.3 (4)
Ru1—Ru2—P2—C13	−20.70 (15)	Ru3—P3—C37—C42	−133.1 (4)
C56—Ru3—P3—C43	−112.4 (2)	C42—C37—C38—C39	0.2 (7)
C57—Ru3—P3—C43	155.4 (2)	P3—C37—C38—C39	179.7 (4)
C55—Ru3—P3—C43	−22.93 (19)	C37—C38—C39—C40	2.3 (8)
Ru2—Ru3—P3—C43	50.91 (19)	C38—C39—C40—C41	−3.4 (8)
Ru1—Ru3—P3—C43	69.20 (16)	C39—C40—C41—C42	2.0 (8)
C56—Ru3—P3—C37	10.4 (2)	C40—C41—C42—C37	0.6 (7)
C57—Ru3—P3—C37	−81.77 (19)	C38—C37—C42—C41	−1.7 (7)
C55—Ru3—P3—C37	99.85 (18)	P3—C37—C42—C41	178.8 (4)
Ru2—Ru3—P3—C37	173.70 (14)	C37—P3—C43—C44	−51.9 (4)
Ru1—Ru3—P3—C37	−168.01 (14)	C30—P3—C43—C44	−157.6 (3)
C56—Ru3—P3—C30	125.4 (2)	Ru3—P3—C43—C44	74.6 (3)
C57—Ru3—P3—C30	33.20 (19)	C37—P3—C43—C48	133.2 (3)
C55—Ru3—P3—C30	−145.18 (19)	C30—P3—C43—C48	27.5 (4)
Ru2—Ru3—P3—C30	−71.34 (18)	Ru3—P3—C43—C48	−100.2 (3)
Ru1—Ru3—P3—C30	−53.05 (15)	C48—C43—C44—C45	−2.7 (6)
C7—P1—C1—C2	128.6 (3)	P3—C43—C44—C45	−177.7 (3)
C13—P1—C1—C2	25.3 (4)	C43—C44—C45—C46	3.1 (6)
Ru1—P1—C1—C2	−100.5 (3)	C44—C45—C46—C47	−1.5 (6)
C7—P1—C1—C6	−54.5 (4)	C45—C46—C47—C48	−0.5 (7)
C13—P1—C1—C6	−157.8 (3)	C46—C47—C48—C43	0.9 (6)
Ru1—P1—C1—C6	76.4 (3)	C44—C43—C48—C47	0.7 (6)
C6—C1—C2—C3	2.4 (6)	P3—C43—C48—C47	175.6 (3)

Symmetry codes: (i) −*x*, −*y*, −*z*+1.

### Hydrogen-bond geometry (Å, °)

**Table:**

Cg1 and Cg2 are the centroids of the C7–C12 and C14–C19 benzene rings, respectively.

**Table:**

*D*—H···*A*	*D*—H	H···*A*	*D*···*A*	*D*—H···*A*
C9—H9A···O1^ii^	0.93	2.53	3.330 (5)	144
C29—H29B···Cg1^iii^	0.96	2.97	3.554 (5)	121
C40—H40A···Cg1^iv^	0.93	2.92	3.670 (6)	139
C58—H58A···Cg2^v^	0.97	2.67	3.585 (19)	158

Symmetry codes: (ii) *x*, −*y*+1/2, *z*+1/2; (iii) *x*+1, *y*, *z*; (iv) −*x*, *y*−1/2, −*z*+1/2; (v) *x*, −*y*−1/2, *z*−1/2.

## References

[bb1] Bruce, M. I., Liddell, M. J., Hughes, C. A., Patrick, J. M., Skelton, B. W. & White, A. H. (1988*a*). *J. Organomet. Chem.* **347**, 181–205.

[bb2] Bruce, M. I., Liddell, M. J., Shawkataly, O. bin, Hughes, C. A., Skelton, B. W. & White, A. H. (1988*b*). *J. Organomet. Chem.* **347**, 207–235.

[bb3] Bruce, M. I., Shawkataly, O. bin & Williams, M. L. (1985). *J. Organomet. Chem.* **287**, 127–131.

[bb4] Bruker (2009). *APEX2*, *SAINT* and *SADABS* Bruker AXS Inc., Madison, Wisconsin, USA.

[bb5] Cosier, J. & Glazer, A. M. (1986). *J. Appl. Cryst.* **19**, 105–107.

[bb6] Filby, M., Deeming, A. J., Hogarth, G. & Lee, M.-Y. (2006). *Can. J. Chem.* **84**, 319–329.

[bb7] Sanger, A. R. (1983). *Can. J. Chem.* **61**, 2214–2219.

[bb8] Shawkataly, O. bin, Khan, I. A., Yeap, C. S. & Fun, H.-K. (2010). *Acta Cryst.* E**66**, m94–m95. 10.1107/S1600536809049927 PMC2980229 21579984

[bb9] Shawkataly, O. bin, Ramalingam, K., Fun, H.-K., Abdul Rahman, A., & Razak, I. A. (2004). *J. Cluster Sci.* **15**, 387–394.

[bb10] Shawkataly, O. bin, Ramalingam, K., Lee, S. T., Parameswary, M., Fun, H.-K. & Sivakumar, K. (1998). *Polyhedron*, **17**, 1211–1216.

[bb11] Sheldrick, G. M. (2008). *Acta Cryst.* A**64**, 112–122. 10.1107/S0108767307043930 18156677

[bb12] Spek, A. L. (2009). *Acta Cryst.* D**65**, 148–155. 10.1107/S090744490804362X PMC2631630 19171970

